# FALL ENCOUNTERS AMONG OLDER ADULTS: IMPACT OF COVID-19

**DOI:** 10.1093/geroni/igad104.2277

**Published:** 2023-12-21

**Authors:** Helen Lach, Joanne salas

**Affiliations:** Saint Louis University, St. Louis, Missouri, United States; Saint Louis University, St. Louis, Missouri, United States

## Abstract

It was suspected that the restrictions in activity and clinical care during the COVID-19 pandemic would result in increased fall risks and falls in older populations. To explore the impact of the pandemic on falls, we examined the trajectory of fall rates among older adults cared for through a large, multi-state, Midwest healthcare system. De-identified medical record data was used to measure falls presenting to emergency, urgent, and hospital care in adults age 50+ from April 2019 through September 2022. We compared the fall rate during the pre-and post-COVID-19 time periods. Differences in fall rates were compared based on age groups, gender, race, and neighborhood socioeconomic status (nSES). The overall fall rate after COVID onset was significantly greater compared to prior to before COVID (11.59; 95%CI:11.50-11.67 vs. 11.20; 95%, CI:11.06-11.33). While White patients had a significantly higher rate of falls post-pandemic, changes in fall rates did not differ based on age, gender, or race. The changes did vary based on nSES, with the lower groups experiencing the largest increases. Findings may be related to differences in access to care and other services both early in the pandemic and as restrictions were lifted. Given the high cost of fall injuries, further work is needed to reduce falls and injuries in older adults.

